# Non-Pharmaceutical Interventions Based on Diet Restriction and Exercise Improve Morphology and Function of Fatty Pancreas in Male *WBN*/*Kob-Lepr* (*Fa*/*Fa*) Rats

**DOI:** 10.3390/ijms27073210

**Published:** 2026-04-01

**Authors:** Kumiko Minato, Yoko Shiroya, Yuka Kurosaka, Hideki Yamauchi, Shigeru Takemori

**Affiliations:** 1 Department of Molecular Physiology, Division of Physical Fitness, The Jikei University School of Medicine, Chofu 182-8570, Japan; shiro.y.109@gmail.com (Y.S.); yamauchi@jikei.ac.jp (H.Y.); sml@jikei.ac.jp (S.T.); 2 Human Ecology, Graduate School, Wayo Women’s University, Ichikawa 272-8533, Japan; 3 Health and Sports Science, Juntendo University, Inzai 270-1695, Japan; y-kurosaka@juntendo.ac.jp; 4 Department of Molecular Physiology, The Jikei University School of Medicine, Tokyo 105-8461, Japan

**Keywords:** diet and exercise, fatty pancreas, inflammation, mitochondria, ultrastructure

## Abstract

Leptin receptor-deficient *WBN*/*Kob-Lepr fa*/*fa* (WKF) rats spontaneously developed chronic pancreatitis and severe diabetes with obesity. Here, we evaluated the protective effects of diet restriction and chronic exercise against fatty and inflammation-induced disorders in the vulnerable pancreas of WKF rats. Six-week-old male WKF rats were divided into obese control (Obese), diet restriction (DR), and diet restriction + exercise (DR + Ex) groups. *WBN*/*Kob* (WK) rats were used as lean control (Lean). Lean and Obese rats had free access to food, whereas food intake for DR and DR + Ex rats was restricted to 69% and 70% of the Obese level, respectively. The DR + Ex rats exercised voluntarily on a wheel ergometer daily. After six weeks, the rats were euthanized with isoflurane after overnight fasting. Obese rats exhibited diabetes, early stages of pancreatitis, diffuse pancreatic islets, and ultrastructural deteriorations in the pancreatic acinar cells, such as lipid droplet accumulation and swollen mitochondria with destroyed cristae, whereas Lean rats did not. DR rats exhibited improved glucose metabolism and serum triglyceride levels, effectively preventing inflammatory processes in the pancreas. However, DR rats exhibited no amelioration in the serum free fatty acids (FFAs) level, and limited improvements in ultrastructural deterioration in pancreatic cells. Chronic exercise combined with diet restriction (DR + Ex) improved serum FFA levels, the boundary of pancreatic islets, and the ultrastructure of subcellular organelles. These results demonstrate that diet restriction suppresses pancreatic inflammation, and further additional exercise effectively improves fatty pancreas-related deterioration by skeletal muscle activity linked through the circulatory network in WKF rats.

## 1. Introduction

Obesity and metabolic syndrome are important health issues. They are commonly accompanied by ectopic fat accumulation in the liver [1], skeletal muscle [2], and pancreas [3]. Fat accumulation in the liver [1] and skeletal muscles [2] functionally impairs insulin resistance, and a fatty pancreas may impair insulin secretion, causing and exacerbating diabetes [4]. Recently, metabolic diseases related to obesity and metabolic syndrome have been named “metabolic dysfunction-associated fatty liver diseases (MAFLDs) [5]” and “metabolic dysfunction-associated fatty pancreas diseases (MAFPDs) [6]”.

In humans with fatty livers, some develop hepatitis with histological inflammatory damage to hepatocytes, which can eventually lead to structural hepatic fibrosis and cirrhosis [5]. Similarly, some patients with fatty pancreas develop pancreatitis with histological inflammatory damage to endocrine islets and exocrine acini [7] and may eventually develop pancreatic cancer [8].

Although excessive lipid deposition can damage subcellular organelles and trigger inflammation, the relationship between the severity of fatty change and the degree of inflammatory response remains unclear. In our previous study, diet restriction, which is considered an effective non-pharmaceutical countermeasure against obesity, was found to markedly accelerate ectopic fat accumulation in the liver of *Zucker Fatty* (ZF) rats [9,10]. Relative starvation with diet restriction accelerates fatty acid synthesis, particularly in the larger fat cells of the adipose tissue, to elevate the plasma levels of free fatty acids (FFAs) [11], which in turn serve as a source of ectopic lipogenesis [12].

Genetically lacking the leptin receptor, ZF rats are considered an extreme leptin resistance model, which has recently attracted attention as a risk factor for human obesity and metabolic syndrome [13]. Leptin secreted from adipose tissue suppresses food intake and facilitates skeletal muscle development [14] and energy expenditure [15]. When freely fed and raised, ZF rats overeat and exhibit poor skeletal muscle development [16]. By 12 weeks of age, ZF rats exhibit insulin resistance, hyperglycemia, hyperlipidemia, and severe ectopic fat accumulation in the liver, skeletal muscles, and pancreas [9,10,17,18,19]. Hepatocytes [10], pancreatic acinar cells [19], and the intermyofibrillar space of poorly developed skeletal muscle cells [17] morphologically deteriorated with excessive fat accumulation and disruption of subcellular organelles.

Diet restriction in ZF rats was found to exacerbate fatty liver, whereas chronic exercise combined with diet restriction was found to markedly improve lipid metabolism, ameliorate ultrastructural abnormalities of subcellular organelles, and reduce ectopic fat accumulation in the liver and pancreas [9,10,18,19]. These findings indicate that impaired pancreatic function could be improved through non-pharmaceutical interventions that include exercise.

In this study, to evaluate the protective effects of non-pharmaceutical countermeasures on fatty and inflammatory disorders of the pancreas, we selected the *WBN*/*Kob-Lepr fa*/*fa* (WK-Fatty; WKF) rat strain [20], which exhibits more severe pancreatic deterioration than ZF rats. The WKF rat strain was developed by introducing a leptin deficiency gene into the original *WBN*/*Kob* (lean) rat [21] genome, which possesses a vulnerable pancreas that spontaneously develops chronic pancreatitis and secondary diabetes. WKF rats tend to overeat and develop obesity, which could induce early onset of diabetes and inflammatory diseases with fatty pancreas [22]. Compared with ZF rats and *db*/*db* mice, which are commonly used obesity and type 2 diabetes models [23], WKF rats exhibit more severe pancreatic inflammatory changes that markedly impair both exocrine and endocrine function, often leading to β-cell failure and severe diabetes development. Therefore, WKF rats may serve as a more appropriate model for MAFPD.

Despite the increasing recognition of MAFPD, the effects of non-pharmaceutical interventions on both exocrine and endocrine pancreatic dysfunction remain poorly understood. In particular, no studies have examined whether combined diet restriction and exercise can ameliorate pancreatic injury in a model that naturally develops pancreatitis, fatty pancreas, and diabetes. Therefore, in this study, we aimed to investigate the protective effects of non-pharmaceutical interventions on pancreatic morphology and function in male *WBN*/*Kob-Lepr* (*fa*/*fa*) rats.

## 2. Results

### 2.1. Animal Characteristics

Compared with lean group (Lean), obese group (Obese) exhibited significantly higher body weight, food intake, and visceral fat (Table 1). With a 15% lower diet intake, diet-restricted group (DR) still accumulated twice the visceral fat with a 7% lower body weight compared with Lean group. Additional chronic exercise in diet-restricted and exercise group (DR + Ex) slightly decreased visceral fat by 10%, while maintaining body weight comparable to that of the DR group.

### 2.2. Glucose and Lipid Metabolism

**Figure 1:**
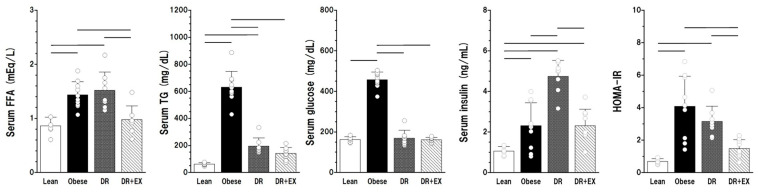
Levels of glucose metabolism (serum glucose, serum insulin, and homeostasis model assessment—insulin resistance; HOMA-IR), lipid metabolism (serum free fatty acid; FFA, serum triglyceride; TG), in Lean, Obese, DR and DR + Ex groups. Means ± SD. Bar represents significant difference (*p* < 0.05). *n* = 6–10 per group.

Glucose and lipid metabolic parameters are shown in Figure 1. Obese group showed severely elevated serum glucose levels and relatively low serum insulin levels, suggesting hyperglycemia with insufficient insulin secretion. Diet restriction improved glucose levels with a marked increase in insulin secretion. The high homeostasis model assessment—insulin resistance (HOMA-IR) of DR indicated persistent insulin resistance, which was remarkably improved by chronic exercise in the DR + Ex group. Obese group also showed a high level of serum triglycerides. Diet restriction in DR markedly improved serum triglycerides but had little effect on the elevated level of serum FFA. Chronic exercise with DR + Ex normalized FFA levels.

### 2.3. Pancreatic Parameters

**Figure 2:**
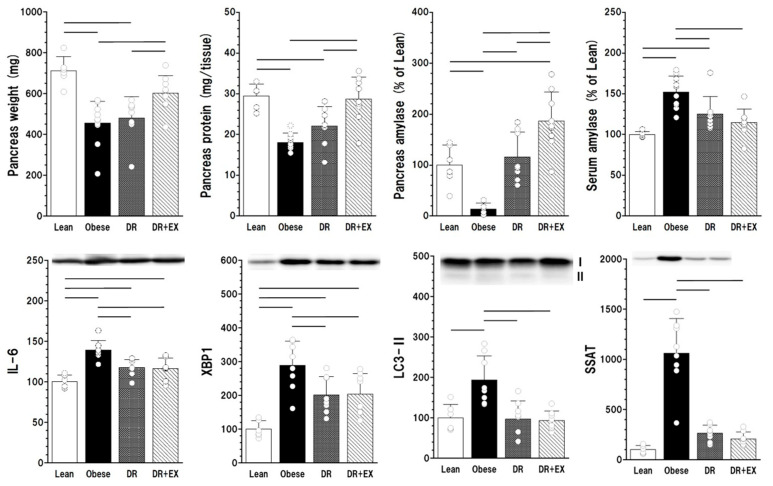
Pancreatic parameters (weight, protein content, amylase level, and serum amylase) and markers of inflammation (interleukin 6, IL-6), endoplasmic reticulum stress (X-box binding protein-1, XBP1), autophagy (microtubule-associated proteins light chain 3-II, LC3-II), and an acute pancreatitis indicator (spermidine/spermine N1-acetyltransferase, SSAT) of the pancreas in Lean, Obese, DR and DR + Ex groups. Means ± SD. Bar represents significant difference (*p* < 0.05). *n* = 6–10 per group.

The parameters related to the state of the pancreas are shown in Figure 2. The weight of the pancreas and pancreatic protein content decreased in Obese group. The pancreatic amylase level decreased to one-fifth of that of Lean group with an increase in the serum amylase concentration, and an acute pancreatitis indicator (spermidine/spermine N1-acetyltransferase, SSAT) increased to ten times the level in Lean group, suggesting ongoing pancreatitis in Obese rats. Diet restriction had no marked effects on the weight and protein content of the pancreas but markedly recovered the pancreatic amylase level with a decrease in serum amylase concentration. As elevated markers of inflammation (Interleukin 6, IL-6), endoplasmic reticulum (ER) stress (X-box binding protein-1, XBP1), autophagy (microtubule-associated proteins light chain 3-II, LC3-II), and SSAT in Obese group were significantly decreased in DR group, inflammatory destruction of pancreatic tissue in Obese rats seemed to be successfully ameliorated in DR. Chronic exercise in the DR + Ex group efficiently recovered the weight, protein content, and amylase levels of the pancreas.

### 2.4. Morphological Observations

#### 2.4.1. Fatty and Inflammatory Deteriorations in Pancreatic Tissues

**Figure 3:**
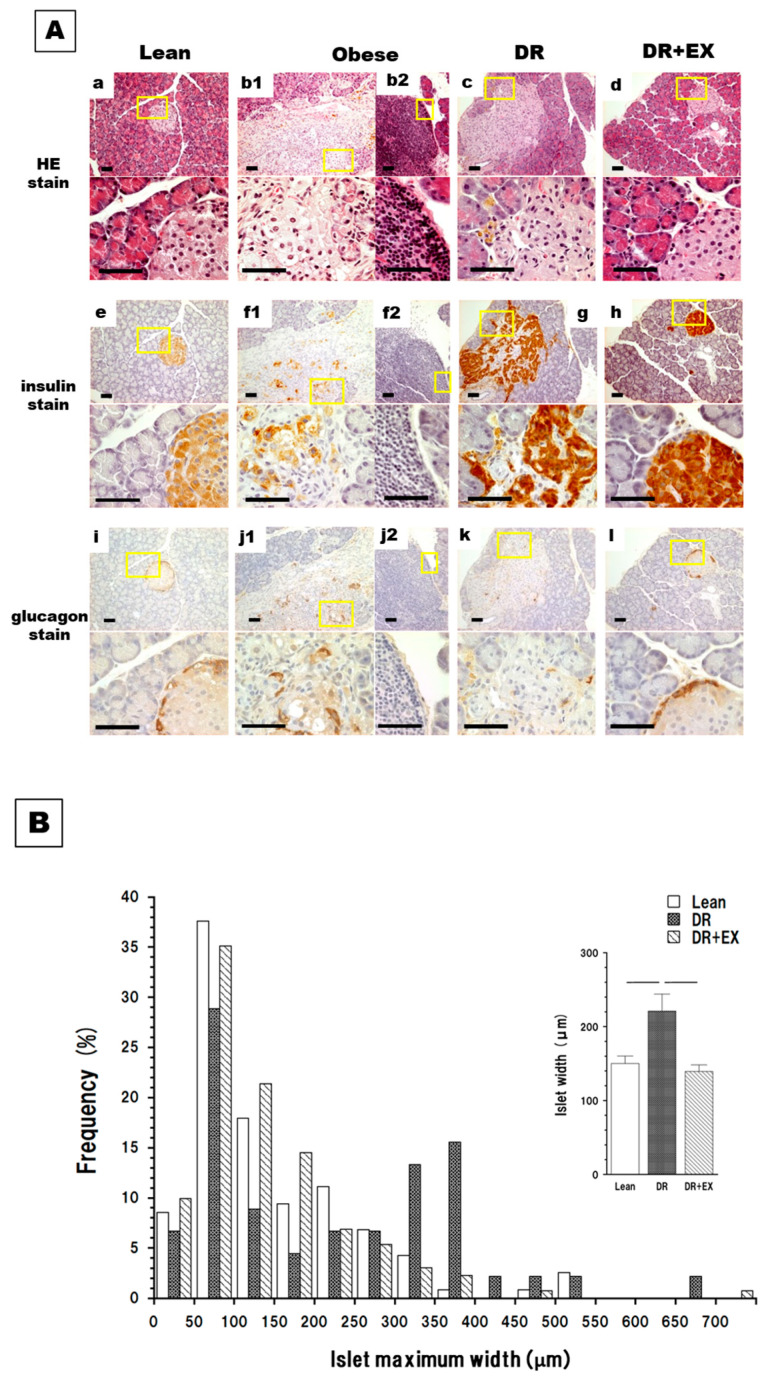
Representative micrographs of pancreatic tissue (**A**) and distribution of maximum width of Langerhans’ islets (LI) (**B**). (**A**): HE- (**a**–**d**), insulin- (**e**–**h**), and glucagon-stained (**i**–**l**) pancreatic tissue in Lean (**a**,**e**,**i**), Obese (**b**,**f**,**j**), DR (**c**,**g**,**k**), and DR + Ex (**d**,**h**,**l**) rats. Pancreatic tissue of Lean rats (**a**) exhibited normal lobular acini and LI with a typical distribution of peripheral glucagon-secreting cells (α-cell; **i**) and central insulin-secreting cells (β-cell; **e**). Pancreatic tissue of Obese rats (**b1**,**f1**,**j1**) showed acinar inflammation as early lesions of pancreatitis with patchy to fairly dense infiltration by inflammatory cells (**b2**,**f2**,**j2**). The boundary between endocrine and exocrine areas was unclear, and the shape of LI was no longer round. Scattered α-cells and β-cells within the expanded abnormal endocrine area in Obese rats (**b1**,**f1**,**j1**) seemed to have fewer β-cells than other rats (**e**,**g**,**h**). Pancreatic tissue of DR rats exhibited almost no signs of inflammation (**c**). In DR + Ex rats, normal pancreatic tissue architecture with nearly normal LI shape was observed (**d**). Bars = 50 µm. Insets are magnified images. (**B**): Distribution of the maximum width of LI in Lean, DR, and DR + Ex groups. Means ± SE. Bars represent a significant difference (*p* < 0.05). *n* = 4–6 per group.

Histological images of the pancreatic tissue are shown in Figure 3A. Lean rats exhibited a normal architecture of lobular acini embedding compact, clearly bordered Langerhans islets (LIs) containing insulin-secreting β-cells and peripheral glucagon-secreting α-cells (Figure 3A(a,e,i)). Obese rats exhibited an extended LI that diffused into the acinar region to blur their boundary and sparsely contained β-cells (Figure 3A(b1,f1,j1)), suggesting a reduction in insulin secretion. Obese rats also exhibited patchy-to-dense inflammatory cell infiltration in the pancreas (Figure 3A(b2,f2,j2)). These findings confirmed that obesity not only leads to fatty pancreas but also pancreatitis in WKF rats. In contrast, DR rats showed almost no sign of pancreatic inflammation and LI had relatively well-defined boundaries (Figure 3A(c,g,k)), suggesting the recovery of the insulin-secreting ability. DR + Ex rats recovered a clear boundary between the LI and acini (Figure 3A(d,h,l)). Figure 3B compares the distribution of the maximal widths of the LI between the Lean, DR, and DR + Ex groups. The areas of the LI in Obese rats were difficult to define for evaluating their maximal widths. The DR group exhibited a significantly larger mean width than other groups. Note the fraction of expanded LIs of maximal width exceeding 300 μm in DR.

#### 2.4.2. Subcellular Fatty Deterioration in Pancreatic Acinar and β-Cells

**Figure 4:**
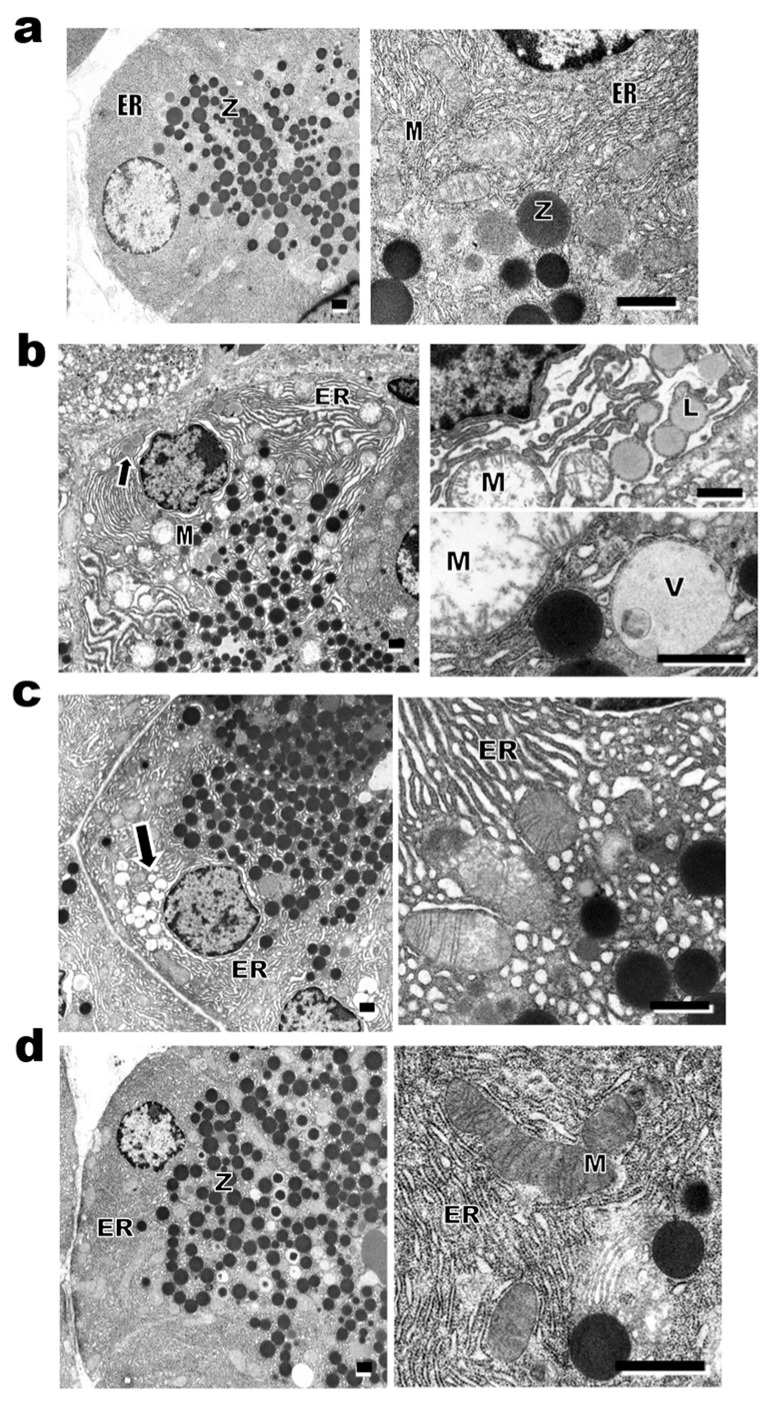
Representative images of electron micrographs of pancreatic acinar cells in Lean (**a**), Obese (**b**), DR (**c**), and DR + Ex (**d**) rats. Lean rats showed normal acinar cells (**a**). The right panels show images of higher magnification. Numerous zymogen granules (Z) consisting of electron-dense material were present in the apical region of the cells approaching the acinar lumen. Well-developed cisternae of rough endoplasmic reticulum (ER) with abundant ribosomes attached to the outer surface of the membrane were observed in the basal region. Oval and rod-like mitochondria (M) with well-developed cristae were present (**a**). Electron micrograph of Obese rats revealed atrophy of the acinar cells, fewer zymogen granules, dilation of rough endoplasmic reticulum (ER), the presence of autophagic vacuole (V), swollen mitochondria (M) with destroyed cristae and accumulation of lipid droplets (arrow, L) in the basal region of the cell (**b**). In DR rats, dilation of rough endoplasmic reticulum (ER) and accumulation of lipid droplets (arrow) were observed (**c**). DR + Ex rats showed a marked increase in zymogen granules (Z), and the ultrastructure of rough endoplasmic reticulum (ER) and mitochondria (M) appeared normal (**d**). Scale bars = 1 μm.

Electron micrographs of the pancreatic acinar cells are shown in Figure 4. The Lean rats showed normal acinar cells with abundant electron-dense zymogen granules in the apical region approaching the acinar lumen. A rough endoplasmic reticulum and mitochondria with well-developed cristae were observed in the basal region (Figure 4a). Obese rats showed fewer zymogen granules, an abnormally dilated rough endoplasmic reticulum, autophagic vacuoles and swollen mitochondria with destroyed cristae (Figure 4b). These findings suggest a reduction in pancreatic protein synthesis. Lipid droplet accumulation was observed in the basal region of the cells (Figure 4b). In the DR rats, dilation of the rough endoplasmic reticulum and accumulation of lipid droplets were observed (Figure 4c). DR + Ex rats showed a marked increase in zymogen granules with a nearly normal rough endoplasmic reticulum and mitochondria, as observed in the Lean rats (Figure 4d).

**Figure 5:**
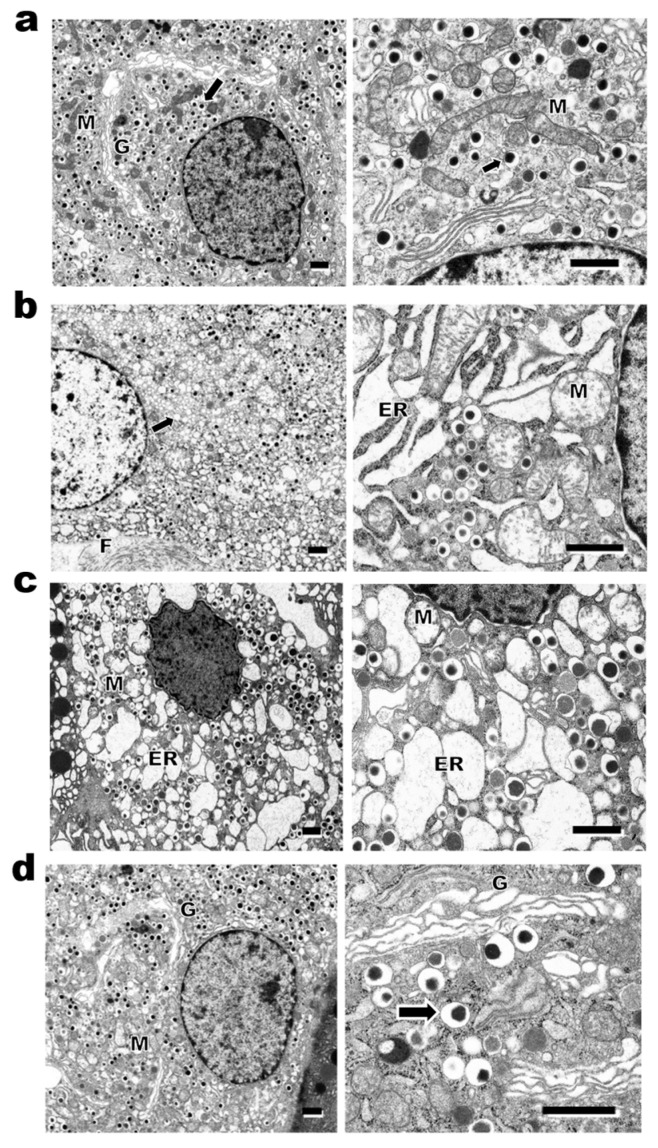
Representative images of electron micrographs of pancreatic β-cells in Lean (**a**), Obese (**b**), DR (**c**) and DR + Ex (**d**) rats. β-cells of Lean rats showed numerous electron-dense secretory granules (arrow) surrounded by a wide electron-lucent halo, large Golgi apparatus (G), and rod-like mitochondria (M) with well-developed cristae (**a**). The right panels show higher magnification. In the β-cells of Obese rats, a decrease and depletion of secretory granules (arrow), dilation of rough endoplasmic reticulum (ER) and swollen mitochondria (M) with destroyed cristae, and collagen fibers (F) adhering to β-cells were observed (**b**). β-cells from DR rats revealed dilation of rough endoplasmic reticulum (ER) and swollen mitochondria (M) (**c**). DR + Ex rats showed obvious improvement in the ultrastructure of β-cells, including increased secretory granules (arrow), mitochondria (M) with well-developed cristae, and well-developed Golgi apparatus (G) similarly to Lean rats (**d**). Scale bars = 1 μm.

Electron micrographs of pancreatic β-cells from Lean rats showed numerous electron-dense secretory granules surrounded by a wide electron-lucent halo, large Golgi apparatus, and rod-like mitochondria with well-developed cristae (Figure 5a). β-cells from Obese rats showed decrease and degranulation of secretory granules, a dilated rough endoplasmic reticulum, and swollen mitochondria with destroyed cristae, indicating a functional decline in β-cells (Figure 5b). Collagen fibers adhering to the β-cells were observed, suggesting a history of inflammation in the LI (Figure 5b). In the DR, a dilated rough endoplasmic reticulum and swollen mitochondria were still present (Figure 5c). DR + Ex rats displayed a clear improvement in the ultrastructure of β-cells, including an increase in secretory granules, a well-developed Golgi apparatus, and mitochondria with well-developed cristae, which appeared similar to those of Lean rats (Figure 5d).

### 2.5. Hepatic Parameters

Hepatic parameters are shown in Supplementary Figure S1. High levels of hepatic triglyceride (TG) and serum alanine transaminase (ALT) in Obese group were observed, and ectopic fat accumulations in the liver were revealed by morphological analysis, suggesting functional damage to the hepatocytes. Diet restriction improved these parameters and chronic exercise combined with diet restriction significantly decreased ectopic fat accumulation in the liver.

### 2.6. Skeletal Muscle Parameters

Atrophy of the soleus, plantaris, and lateral gastrocnemius (LG) muscle was observed in Obese rats (Supplementary Figure S2). DR group showed lower soleus and plantaris muscle weight than Obese group. Chronic exercise with DR + Ex increased muscle weight but barely reached the Lean level. The levels of glucose transporter 4 (GLUT4), peroxisome proliferator-activated receptor gamma coactivator 1(PGC1), and cytochrome c oxidase IV (COX IV) were lower in Obese than in Lean group, indicating glucometabolic and mitochondrial inactivity in the soleus muscle (Supplementary Figure S3). Glucometabolic (GLUT4, hexokinase 2 (HK2), and AMP-activated protein kinase (AMPK)) and mitochondrial (citrate synthase, CS and COX IV) indicators in both muscles were highest in the DR + Ex group (Supplementary Figure S3). Representative sections from LG muscle stained with cytochrome oxidase demonstrate these changes in COX IV level and a prominent increase in darkly stained fiber in the DR + EX rats (Supplementary Figure S2), which is an effect of chronic exercise on glucometabolic function observed in muscles, especially in fast-twitch muscle.

Electron micrographs of the soleus muscle fibers from Lean rats appeared normal with intact mitochondria (Supplementary Figure S4a). Obese rats exhibited ectopic deposition of lipid droplets and prominent mitochondrial swelling (Supplementary Figure S4b). Rod-like mitochondrial swelling was observed in DR rats (Supplementary Figure S4c). In DR + Ex rats, intracellular lipid droplets were markedly reduced, and most mitochondria were restored to their normal shape, as observed in Lean rats (Supplementary Figure S4d).

## 3. Discussion

### 3.1. Early Inflammation in the Fatty Pancreas

The fatty pancreas of Obese rats histologically (Figure 3) and biochemically (Figure 2) exhibited early stage of pancreatitis development at 12 weeks of age. Inflammatory disruption of the pancreatic endocrine structure exacerbated insulin secretion, resulting in severe insulin-deficient hyperglycemia.

Diet restriction, which suppressed body weight gain, had no ameliorating effect on the serum FFA levels and visceral fat mass (Figure 1), and limited ameliorating effects on lipid droplet deposition, ultrastructural deterioration of subcellular organelles in pancreatic cells (Figure 4 and Figure 5), and skeletal muscle fibers (Supplementary Figure S4). This may reflect impaired FFA utilization in the atrophied skeletal muscles of DR rats (Figure 2). Nevertheless, diet restriction effectively diminished the inflammatory processes in the pancreas. Endocrine function was preserved, as evidenced by the insulin-rich normal serum glucose level in DR rats (Figure 1). Inflammation, ER stress, and autophagy markers, which were elevated in the pancreas of Obese rats, were improved in DR rats (Figure 2). Furthermore, polyamine catabolic enzyme, recognized as an acute pancreatitis indicator [24], was significantly increased in Obese group and decreased in DR group to the same level as that of Lean group (Figure 2). These findings suggest that, although leptin resistance may have contributed to the induction of early inflammatory responses in obese WKF rats, lipid deposition and deterioration of subcellular organelles under high-FFA conditions in DR did not exacerbate inflammation. Calorie restriction without malnutrition, exerts a potent anti-inflammatory effect [25]; therefore, it is likely that diet restriction effectively suppressed the pancreatic inflammatory changes associated with obesity-induced overnutrition.

To reduce FFA levels and improve the morphology of subcellular organelles, chronic exercise in combination with diet restriction was necessary, which is consistent with our previous findings in ZF rats [10,19]. The distinct outcomes observed between diet restriction alone and diet restriction plus chronic exercise may reflect a fundamental distinction between simple fatty changes and inflammatory processes. That is, the fatty deterioration of lipid deposition and disruption of subcellular organelles is not inevitably accompanied by inflammation.

### 3.2. Inflammation, ER Stress, and Autophagy

In chronic pancreatitis, inflammatory markers such as IL-6 and TNF-α are elevated in the pancreas and are implicated in its development [26]. Similar increases in these pro-inflammatory markers have been reported in rats fed a high-fat diet; however, 8-week swimming training was found to suppress these elevations, demonstrating the anti-inflammatory effects of exercise [27]. The IL-6 findings in this study are consistent with these observations.

As pancreatic exocrine and endocrine cells have high protein synthesis demands, they possess a well-developed endoplasmic reticulum (ER) and are therefore susceptible to ER stress caused by abnormal proteins accumulations. When autophagy is impaired, ER stress is exacerbated, leading to inflammation and cellular dysfunction [28]. In obesity and metabolic disorders, chronic nutrient excess persistently activates adaptive stress response pathways; however, ER homeostasis is often not restored, resulting in inflammation and endocrine dysregulation [29]. In this study, the elevated autophagy, ER stress, and inflammatory markers observed in Obese rats were consistent with these mechanisms, suggesting that autophagy activation may have been insufficient for removing abnormal proteins and suppressing ER stress.

The ER and mitochondria are physically and functionally interconnected, and their abnormalities in metabolic diseases mutually aggravate one another [30], promoting pancreatic inflammation and pancreatitis [31]. Glucolipotoxicity in obesity and diabetes is known to induce mitochondrial dysfunction in β-cells [32], and similar abnormalities were observed in pancreatic acinar cells, β-cells, and soleus muscle fibers in this study. Exercise has been reported to protect β-cells from ER stress and apoptosis [33] and enhance mitochondrial biogenesis [34]. In *db*/*db* mice, which develop obesity, diabetes, and fatty liver similar to WKF rats, exercise improves glucose and lipid metabolism by alleviating oxidative and ER stress, and activating antioxidant and AMPK-related pathways as effectively as metformin [35].

In this study, chronic exercise produced clear beneficial effects on metabolic parameters and the ultrastructure of pancreatic cells. However, no additive effects of combining exercise with diet restriction were observed for inflammatory, ER stress, or autophagy markers, likely because inflammation had already been sufficiently suppressed by diet restriction alone. The mechanisms underlying these findings remain unclear, and further detailed investigations using a broader range of molecular indicators are required warranted because the markers analyzed in this study were limited.

### 3.3. Effects of Chronic Exercise

To maintain physiological pancreatic endocrine and exocrine functions, chronic exercise to reduce elevated FFA levels in combination with diet restriction is necessary [11]. Skeletal muscle is the only organ that can be voluntarily activated. Chronic exercise increases the mitochondrial volume and function in muscle tissue [36], induces muscle hypertrophy [37], and enhances glucose and FFA utilization [38,39,40].

Peroxisome proliferator-activated receptor gamma (PPARγ) ligands prevent chronic pancreatitis in WBN/Kob rats [41]. Thiazolidinedione derivatives (TZDs), which are PPARγ agonists, are clinically used to treat type 2 diabetes [42] because they reduce insulin resistance, exert anti-inflammatory effects, and improve lipid metabolism. Aerobic exercise also activates PPARγ-PGC1α signaling in skeletal muscle, thereby increasing mitochondrial density and function [36].

In our observations (Supplementary Figure S3), insulin sensitivity [43], Glut4- and AMPK-mediated glucose uptake [38,44] and mitochondria function (PGC1) [45] were only partially ameliorated in DR rats but were significantly improved in DR + EX rats. Mitochondrial morphological improvements were observed in pancreatic acinar and β-cells as well as in skeletal muscle cells, suggesting that enhanced mitochondrial function via the PPARγ–PGC1α pathway may contribute to these beneficial effects.

The DR pancreas exhibited a fraction of diffusely enlarged LIs (Figure 3B). Although enlargement itself did not promote inflammation, it may provide sites vulnerable to attacks leading to inflammation. One of the possible vulnerable sites is the islet–exocrine interface (IEI), which contains pericytes similar to vascular endothelial cells [46], a known target of inflammation in metabolic syndromes, such as diabetes. In this study, substantial improvement in the IEI structure and numerous compact LIs with well-defined physiological IEI were observed in the DR + EX group (Figure 3A), indicating that exercise contributed to structural stabilization.

### 3.4. Exercise and Exocrine Pancreatic Function

Various beneficial effects of exercise on exocrine pancreatic function have been reported. Exercise enhances this function in healthy rats [47,48,49,50,51,52]; for instance, endurance running increases pancreatic enzymes secretion in response to cholecystokinin (CCK) [51], and induces hypertrophy of the acinar pancreas of zymogen-rich cells [50,52]. The beneficial effects of exercise have been found to disappear with CCK antagonists [53] or in vagotomized rats [47], indicating the involvement of both endocrine and neural regulation.

Pancreatic protein content and enzyme activities are indicators of digestive enzyme synthesis and storage. Aging reduces pancreatic amylase reserves and attenuates enzyme secretory response to stimulation [54]. These parameters also decline with pancreatic atrophy with obesity and diabetes [55,56]. Our previous study demonstrated that age-related and obesity-induced reductions in exocrine pancreatic function have been shown to be reversed by chronic exercise [18,19,57].

In this study, the Obese group exhibited marked decreases in pancreatic weight, pancreatic protein content, and pancreatic enzyme levels, indicating exocrine pancreatic dysfunction, a characteristic feature of pancreatitis. Diet restriction alone produced only modest improvement, whereas the addition of chronic exercise resulted in significant recovery. Elbassuoni et al. [58] reported that chronic swimming exerts anti-inflammatory and antioxidant effects that protect the pancreas from immobilization-induced inflammation. Although our study demonstrated that diet restriction alone was sufficient to prevent early pancreatic inflammation in WKF rats, we cannot rule out the additional anti-inflammatory and antioxidant effects of chronic exercise [59,60]. Notably, improvements were observed not only in endocrine function but also in exocrine function, even in pancreata exhibiting inflammatory deterioration.

In our previous study, voluntary running was more effective than compulsory running in promoting exocrine function [50]. From the perspective of skeletal muscle activation [37] and visceral blood flow [61], the chronic exercise applied in this study likely corresponded to voluntary moderate-intensity aerobic activity. Such aerobic exercise enhances vascular function [62] and increases splanchnic blood flow [63], which may improve CCK sensitivity and stabilize vagal nerve activity. These circulatory effects, together with enhanced metabolic and mitochondrial function, could contribute to the superior exocrine pancreatic function observed in the DR + EX group. Importantly, adding chronic exercise to diet restriction did not worsen inflammation and reduced ectopic fat accumulation while improving tissue architecture and subcellular organelles, particularly mitochondrial structure.

In summary, diet restriction improved inflammation by reducing caloric intake; however, it did not enhance FFA metabolism or mitochondrial function, likely because reduced caloric intake led to decreased muscle mass and consequently decreased FFA utilization capacity. In contrast, the addition of chronic exercise resulted in clear improvements in metabolic abnormalities and exocrine pancreatic function, accompanied by restored pancreatic structure and function, particularly those of the mitochondria. These results suggest that daily physical activity combined with a healthy diet to maintain healthy body weight may help to preserve pancreatic health in metabolic disorders, such as diabetes, nonalcoholic fatty pancreas disease (NAFPD) [7,8], and MAFPD [6]. Chronic exercise may therefore provide additional benefits beyond diet restriction alone and support the potential role of non-pharmaceutical interventions, including exercise therapy, in metabolic diseases accompanied by inflammation.

### 3.5. Limitations of This Study

This study has several limitations. The findings were derived from a 6-week intervention in young rats, and neither long-term effects nor outcomes in middle-aged or older animals were assessed. Owing to strain characteristics, female rats and an exercise-only group could not be included; females have a low incidence of pancreatitis, and genetically obese males exhibit severe muscle atrophy and extremely low spontaneous activity. Therefore, the findings of this work should be interpreted within the context of this specific model with a minimal number of animals; direct extrapolation to humans is not possible. Further work should also evaluate different exercise modalities, intervention durations, and animal characteristics to better determine the therapeutic potential of non-pharmaceutical interventions.

## 4. Materials and Methods

### 4.1. Animals

All experiments were approved by the experimental animal ethical committee at Wayo Women’s University (No. 1214), and rats were maintained in accordance with the “Standards Relating to the Care and Keeping and Reducing Pain of Laboratory Animals” of the Ministry of the Environment of Japan.

Male *WBN*/*Kob-Lepr fa*/*fa* (WKF) and *WBN*/*Kob* (WK) rats (age, 5 weeks) were purchased from Sankyo Labo Service Corporation (Tokyo, Japan). After a week of pre-breeding, WKF rats (age, 6 weeks) were allocated into obese (Obese; *n* = 10), diet restricted (DR; *n* = 8), and diet restricted plus chronically exercised (DR + Ex; *n* = 9) groups using stratified allocation based on initial body weight to ensure comparable baseline characteristics. The WKF rats were divided into 3 groups at the beginning of the experimental period (6 weeks of age), ensuring that the mean of the initial body weight was equivalent among the groups. WK rats (age, 6 weeks) were used as a lean control (Lean; *n* = 6). These rats were maintained in individual cages under a 12 h day–night cycle in a temperature-controlled (21 ± 1 °C) room.

### 4.2. Diet

All rats had free access to water. Body weight and food intake were measured daily. Lean and Obese rats had free access to standard chow (NMF; Oriental Yeast, Tokyo, Japan). DR rats received 70% of the average daily food intake of Obese rats, whereas DR + Ex rats were pair-fed to match the body weight of DR rats. Specifically, the daily food amount for each rat in DR and DR + Ex was adjusted based on the Obese rats’ intake on the previous day and the individual body weight on the current day. Eventually, DR and DR + Ex rats had mean food intake levels of 69% and 70% of the Obese rats, respectively. This dietary procedure followed the methods of our previous study [9].

### 4.3. Exercise

The Lean, Obese, and DR rats were kept sedentary during the experimental period. The DR + Ex rats were exercised voluntarily on the wheel ergometer with a load of 30% of their body weight daily [37]. The mean running distance was 1711 ± 458 m/day.

### 4.4. Sampling

Following the 6-week experimental period, the rats were euthanized with isoflurane after an overnight fast. Blood was collected from the abdominal vein, and the pancreas, liver, visceral fat, and hindlimb plantar flexor muscles were rapidly excised and weighed. All pancreatic sampling was performed by the same preclinical pancreatic researcher following a standardized protocol to ensure consistent tissue collection. To reduce potential confounding factors, all sampling was performed under overnight-fasted conditions, within the same experimental period, and at the same time of day.

### 4.5. Determinations

The primary outcomes of this study were morphological analysis of the pancreas, metabolic parameters related to glucose and lipid metabolism, and exocrine pancreatic function. Secondary outcomes included protein expression levels assessed by Western blotting.

Serum TG, glucose, ALT, and amylase were measured by DRY-CHEM 4000 (FUJIFILM Wako Pure Chemical, Tokyo, Japan) and FUJI DRY-CHEM slides (FUJIFILM Wako Pure Chemical, Tokyo, Japan). Serum insulin was measured using an enzyme-linked immunosorbent assay kit (Morinaga Institute of Biological Science, Tokyo, Japan). Serum FFA was measured using a NEFA C-test Wako kit (Wako Pure Chemical Industries. Osaka, Japan). Homeostasis model assessment—insulin resistance (HOMA-IR) was calculated as the serum glucose level (mmol/L) × serum insulin level (µU/mL)/405.

A portion of the pancreas (approx. 50 mg) was homogenized in 10 volumes of saline using a Polytron^®^-homogenizer (Kinematica AG, Malters, Switzerland), and centrifuged at 9000 rpm (8720× *g*) for 30 min at 0 °C. The supernatant was collected to analyze protein content and amylase activity. The pancreatic protein content was measured using the Lowry’s method. Pancreatic amylase was measured using an enzymatic kit (Bioo Scientific, Austin, TX, USA) for colorimetric analysis.

The resected liver was homogenized to extract lipids with a 2:1 chloroform–methanol mixture (*v*/*v*). Hepatic TG was measured using the TG E Test (Wako Pure Chemical Industries, Osaka, Japan).

### 4.6. Western Blotting

The pancreas, and soleus, and plantaris muscles were homogenized with an ultra-turrax homogenizer for 30 s in ice-cold RIPA buffer containing protease and phosphatase inhibitor cocktails (Sigma-Aldrich Japan, Tokyo, Japan). The homogenates were incubated for 1 h at 4 °C on a rotator, followed by centrifugation for 15 min at 16,000× *g*. The protein concentration of the supernatant was measured using Lowry’s method. Protein from each sample was run through SDS-PAGE and immunoblotted according to standard procedures. The immunoblots were analyzed using enhanced chemiluminescent reagent (ImmunoStar Zeta or LD, FUJIFILM Wako Pure Chemical, Tokyo, Japan) in an imaging apparatus (LAS-3000mini, FUJIFILM Wako Pure Chemical, Tokyo, Japan) and were analyzed using Multi Gauge software 3.1(FUJIFILM Wako Pure Chemical, Tokyo, Japan).

### 4.7. Antibodies

The following primary antibodies were used: rabbit anti-IL-6 (Abcam (Cambridge, UK), 6672), rabbit anti-XBP1 (Abcam, 37152), rabbit anti-LC3 (Cell Signaling (Danvers, MA, USA), 12741), rabbit anti-(SSAT1 (Proteintech (Rosemont, IL, USA), 10708-1-AP), rabbit anti-Glut4 (Millipore (Burlington, MA, USA), 07-1404), rabbit anti-HK2 (Millipore, AB3279), rabbit anti-CS (Cell Signaling, 14309), rabbit anti-COX IV (Cell Signaling, 4850), phosphorylated AMPKα Thr172 (Cell Signaling, 2535), and rabbit anti-PGC1 (Santacruz (Santa Cruz, CA, USA), SC-13067). Secondary horseradish peroxidase-linked anti-rabbit IgG (Cell Signaling, 7074) antibody was used.

### 4.8. Morphological Analysis

Other portions of the pancreatic and hepatic tissues were fixed in 10% formalin. Hematoxylin and eosin (HE) staining was outsourced to SRL (Tokyo, Japan). Additionally, pancreatic insulin and glucagon immuno-staining was performed at SRL. Histological and immunohistochemical staining were also outsourced to SRL and performed without knowledge of group allocation. Morphometric analysis was performed using RS Image 1.9.2 software (Roper Scientific, Trenton, NJ, USA) and ImageJ 1.46r [64]. Moreover, samples of the pancreas and soleus muscle tissues were fixed with 2.5% glutaraldehyde in 0.1 M cacodylate buffer at pH 7.4, postfixed with 1% osmium tetroxide in the same buffer, dehydrated, and embedded in Epon 812. Thin sections were cut, stained with uranyl acetate and lead citrate, and examined with a JEOL 1220 electron microscope (JEOL, Tokyo, Japan). The negative film of the electron micrographs was scanned using a scanner (GT-9800F and EPSON Scan 3.04J software, Seiko Epson, Nagano, Japan). To assess the oxidative muscle fiber distribution, serial cross-sections (thickness 10 μm) of LG muscle belly were cut using a cryo-microtome and stained enzyme-histochemically with cytochrome oxidase following a standard procedure. The stained section was captured using a microscope BZ-9000 (Keyence, Osaka, Japan) equipped with a personal computer. The acquired images were used for morphometric analysis.

### 4.9. Statistical Analyses

Data were expressed as means ± SD, except for the islet width, which was expressed as means ± SE. Comparisons between the Lean and other groups were performed using the Dunnett test in StatView software 5.0 (Hulinks, Tokyo, Japan). Multiple comparisons among three groups of the Obese, DR, and DR + Ex rats were performed using the Games–Howell test in the same software. Statistical significance was identified at the 0.05 level.

## 5. Conclusions

Our study on WKF rats indicated the following:

(1) Obesity caused by leptin resistance led to the early onset of pancreatic inflammation and diabetes.
(2) Diet restriction suppressing weight gain of the animal only partly ameliorated lipid accumulation in the pancreas but effectively suppressed pancreatic inflammation. Fatty deterioration is not invariably accompanied by inflammation.
(3) Chronic exercise combined with diet restriction was necessary to preserve serum FFA levels and substantially reduce intracellular ectopic fat accumulation, further improving the cellular architecture and ultrastructure of subcellular organelles of the pancreas.

These findings suggest that chronic exercise combined with diet restriction could preserve both endocrine and exocrine pancreatic function in metabolic disorders.

## Figures and Tables

**Table 1:** Physiological characteristics of rats.

	*WBN*/*Kob*-Lean	*WBN*/*Kob-Fatty*		
	Lean (*n* = 6)	Obese (*n* = 10)	DR (*n* = 8)	DR + EX (*n* = 9)
Body Weight, g	284.1 ± 9.5	333.8 ± 14.9 ^a^	265.4 ± 4.9 ^a,b^	263.7 ± 5.8 ^a,b^
Food Intake, g/day	19.0 ± 1.0	23.2 ± 1.9 ^a^	16.0 ± 0.2 ^a,b^	16.3 ± 0.4 ^a,b^
Visceral Fat Weight, g	3.48 ± 0.62	7.51 ± 0.61 ^a^ (*n* = 8)	6.87 ± 0.65 ^a^ (*n* = 6)	6.06 ± 0.94 ^a,b,c^

Values indicate means ± SD; The Dunnett test compared Lean and Fatty 3 groups. Multiple comparisons among the Fatty 3 groups using Games–Howell test, ^a^: *p* < 0.05, Lean; ^b^: *p* < 0.05, Obese; ^c^: *p* < 0.05, Diet Restriction.

## Data Availability

The original contributions presented in this study are included in the article/Supplementary Material. Further inquiries can be directed to the corresponding author.

## Supplementary Materials

The following supporting information can be downloaded at: https://www.mdpi.com/article/10.3390/ijms27073210/s1.

## Abbreviations

The following abbreviations are used in this manuscript:

WBN/Kob	Wistar Bonn/Kobori
WK	Wistar Bonn/Kobori
WKF	Wistar Bonn/Kobori fatty
ZF	Zucker fatty
DR	Diet restriction
EX	Exercise
FFA	Free fatty acid
NAFPD	Non-alcoholic fatty pancreas disease
MAFPD	Metabolic dysfunction-associated fatty pancreas disease
MAFLD	Metabolic dysfunction-associated fatty liver disease
HOMA-IR	Homeostasis model assessment—insulin resistance
LI	Langerhans’ islets
IEI	Islet–exocrine interface
HE	Hematoxylin and eosin
ER	Endoplasmic reticulum
TG	Triglyceride
ALT	Alanine transaminase
IL6	Interleukin 6
XBP1	X box-binding protein 1
LC3	Microtubule-associated proteins light chain 3
SSAT1	Spermidine/spermine N1-acetyltransferase 1
Glut4	Glucose transporter 4
HK2	Hexokinase 2
CS	Citrate synthase
COX IV	Cytochrome c oxidase IV
AMPKα	AMP (Adenosine monophosphate)-activated protein kinase α
PGC1	Peroxisome proliferator-activated receptor gamma coactivator 1
IgG	Immunoglobulin G
LG	Lateral gastrocnemius
SD	Standard deviation
SE	Standard error
