# Supplementary material for: Non-Pharmaceutical Interventions Based on Diet Restriction and Exercise Improve Morphology and Function of Fatty Pancreas in Male WBN/Kob-Lepr (Fa/Fa) Rats
Source: Int J Mol Sci. 2026 Apr 1;27(7):3210. doi: 10.3390/ijms27073210 (PMC13072781; doi:10.3390/ijms27073210)
Supplement: Supplementary file 1 [file ijms-27-03210-s001.zip › supplementary figure legend.pdf]

Figure Legends (Supplementary figures)

Figure S1. Hepatic weight, serum alanine transaminase (ALT) concentration, and representative micrographs of hepatic tissue stained with HE. Bars represent significant difference ($p<0.05$). *n*=6-10 per group. Scale bars = 50μm.

Figure S2. Skeletal muscle weight and representative cross-sections of lateral gastrocnemius (LG) muscle stained with cytochrome oxidase. Bars represent significant difference ($p<0.05$). *n*=6-10 per group.

Figure S3. glucose transporter 4 (GLUT4), hexokinase 2 (HK2), AMP-activated protein kinase (AMPK) aThr172, peroxisome proliferator-activated receptor gamma coactivator 1(PGC1), citrate synthase (CS), and cytochrome c oxidase IV (COX IV) in soleus and plantaris muscles. Means ± SD. Bars represent significant difference ($p<0.05$). *n*=6-9 per group.

Figure S4. Representative images of electron micrographs of the soleus muscle fibers.
Soleus muscle fibers from Lean rats appeared normal with intact mitochondria (a). In Obese rats, ectopic deposition of lipid droplets (L) and mitochondrial swelling (arrow) were prominent (b). In DR rats, few lipid droplets and rod-like mitochondrial swelling (arrows) were observed (c). In DR+Ex rats, intracellular lipid droplets were markedly reduced, and mitochondria were restored to their normal shape (d). Scale bars = 1μm.
