# Supplementary figures and images for: Non-Pharmaceutical Interventions Based on Diet Restriction and Exercise Improve Morphology and Function of Fatty Pancreas in Male WBN/Kob-Lepr (Fa/Fa) Rats
Source: Int J Mol Sci. 2026 Apr 1;27(7):3210. doi: 10.3390/ijms27073210 (PMC13072781; doi:10.3390/ijms27073210)

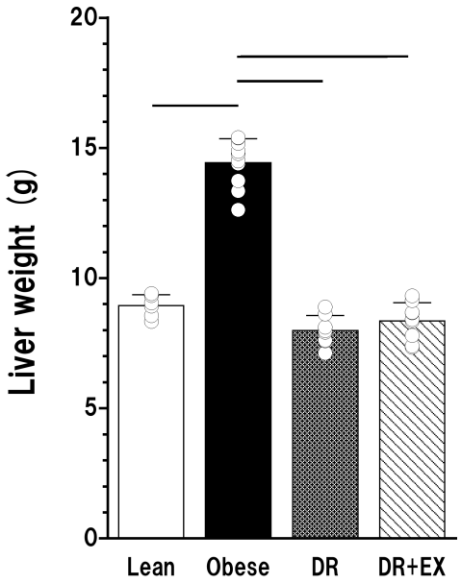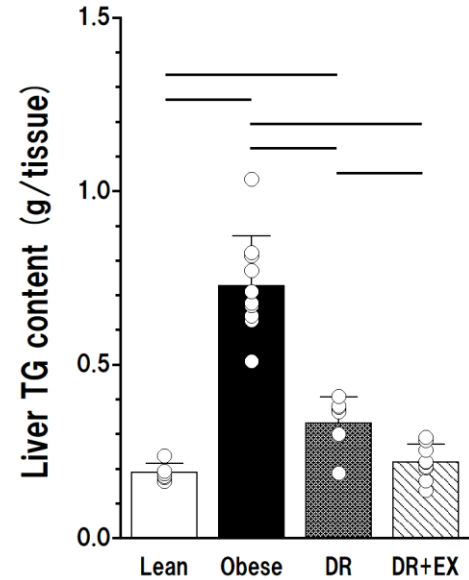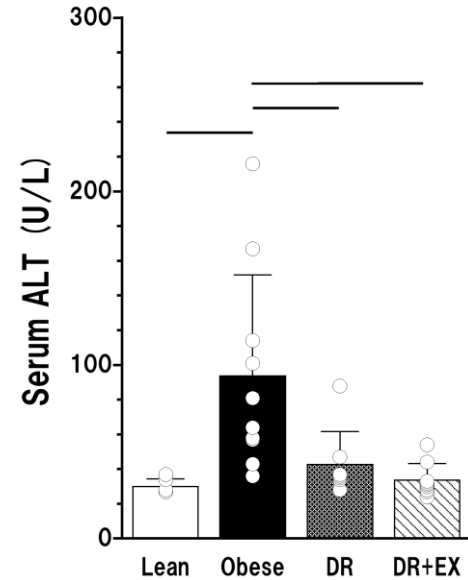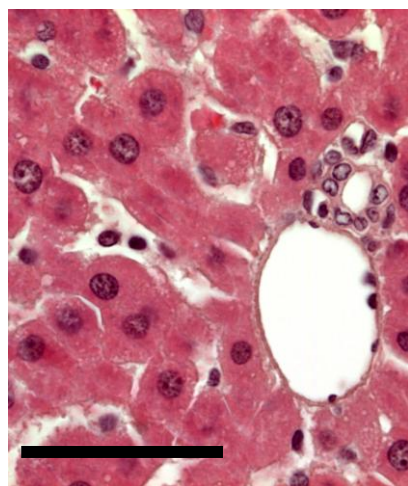

Lean

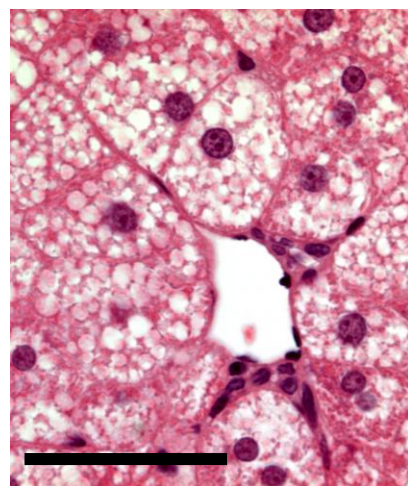

Obese

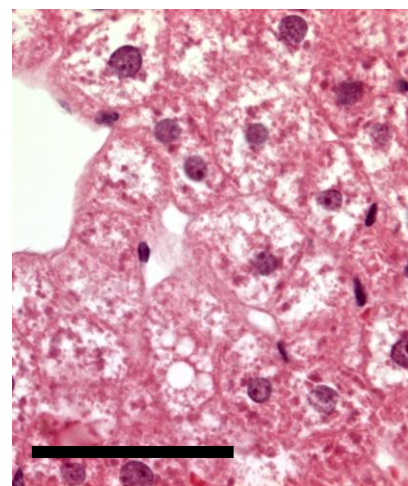

DR

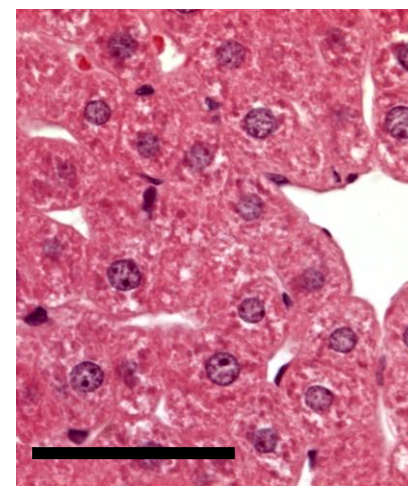

DR + EX

Supplement: Supplementary file 1 [file ijms-27-03210-s001.zip › Supplementary Figure S1.pdf]

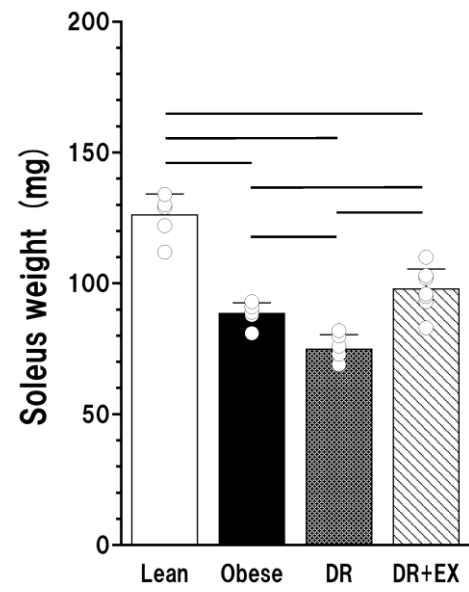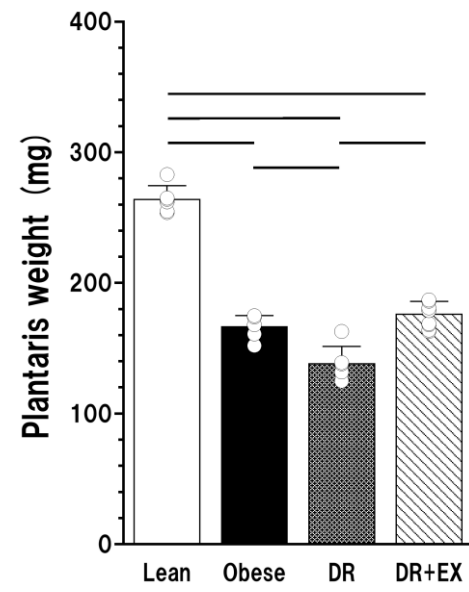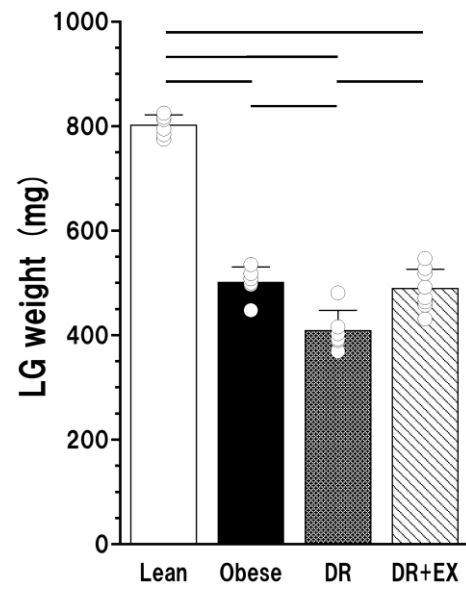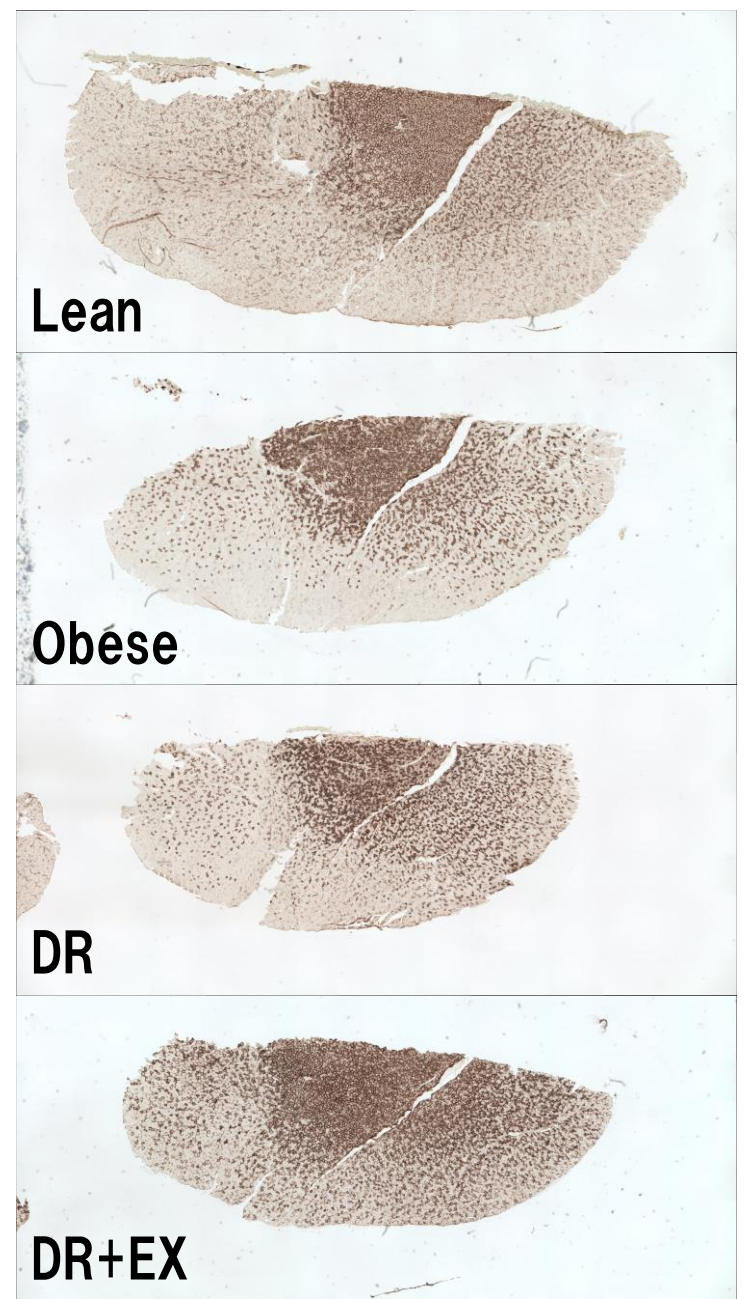

Supplement: Supplementary file 1 [file ijms-27-03210-s001.zip › Supplementary Figure S2.pdf]

# Soleus

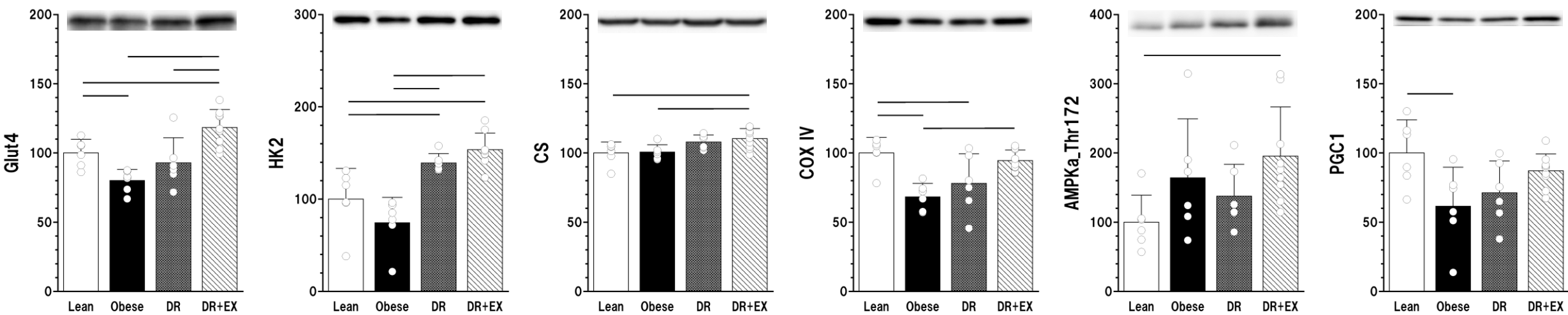

# Plantaris

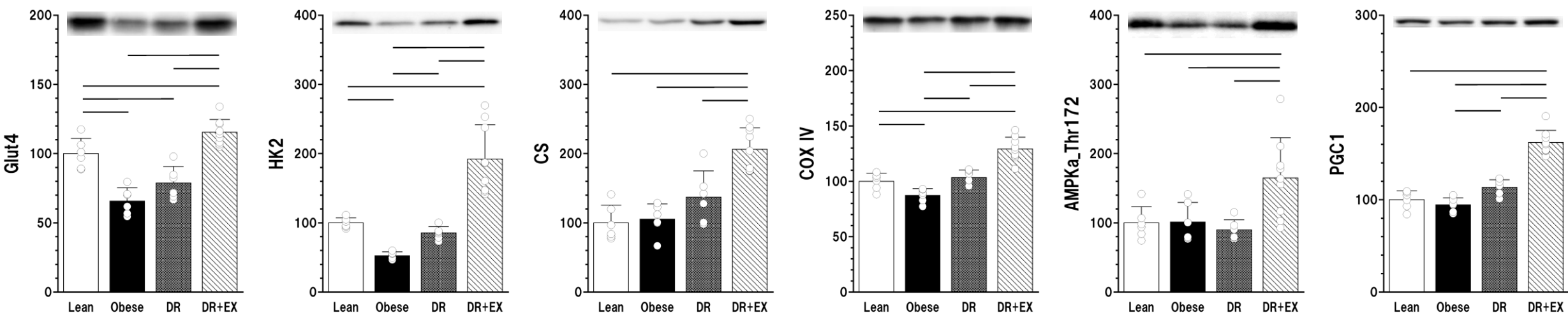

Supplement: Supplementary file 1 [file ijms-27-03210-s001.zip › Supplementary Figure S3.pdf]

**a**

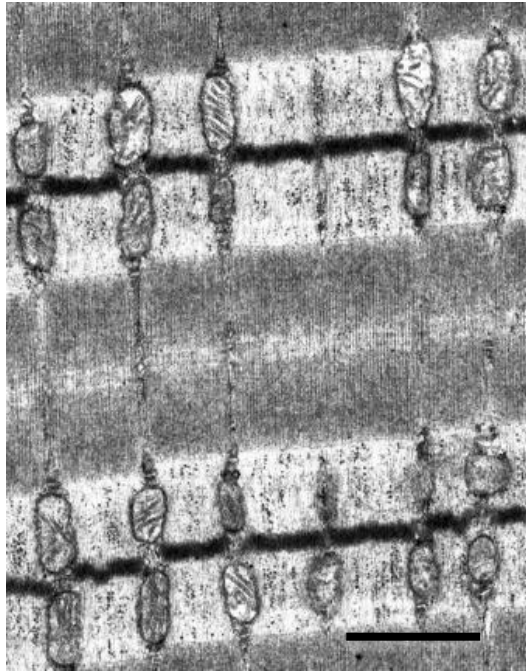

Lean

**b**

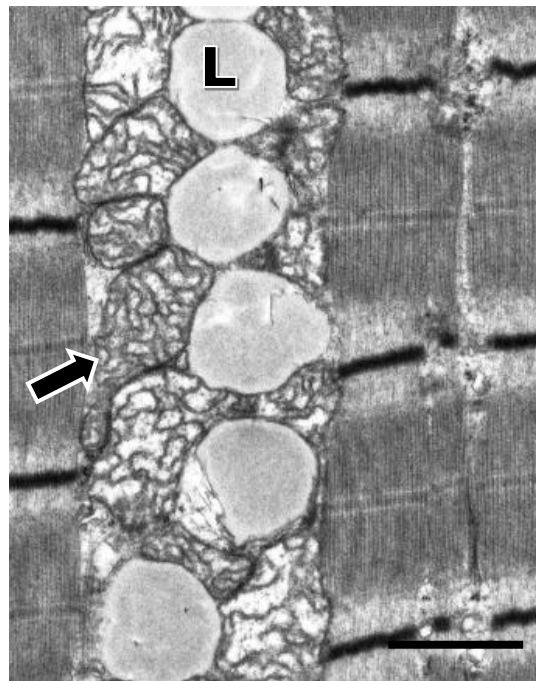

Obese

**c**

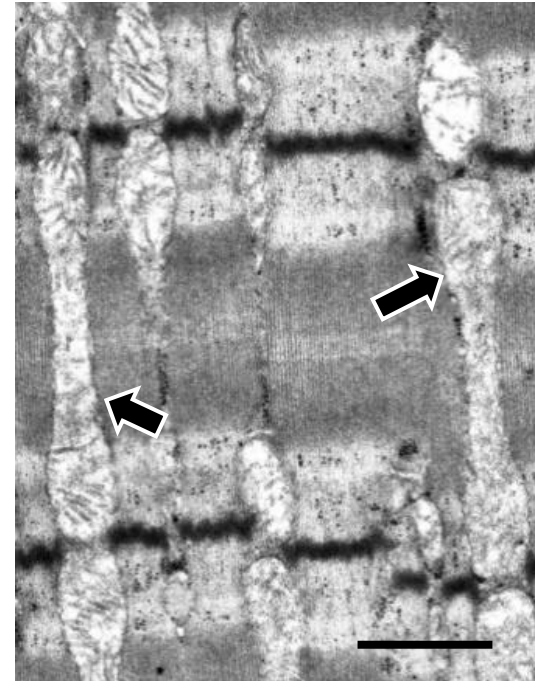

DR

**d**

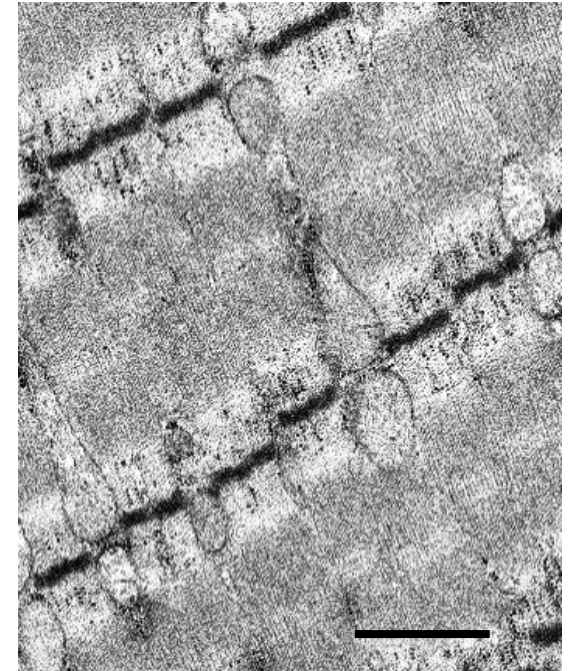

DR + EX

Supplement: Supplementary file 1 [file ijms-27-03210-s001.zip › Supplementary Figure S4.pdf]
